# Supplementary material for: Ataxia-linked SLC1A3 mutations alter EAAT1 chloride channel activity and glial regulation of CNS function
Source: J Clin Invest. 2022 Apr 1;132(7):e154891. doi: 10.1172/JCI154891 (PMC8970671; doi:10.1172/JCI154891)
Supplement: Supplemental data [file jci-132-154891-s192.pdf]

Supplementary materials for

Ataxia-linked SLC1A3 mutations alter EAAT1 chloride channel activity and glial regulation of CNS function

Qianyi Wu[1#], Azman Akhter[2#], Shashank Pant[3], Eunjoo Cho[4], Jin Xin Zhu[4], Alastair Garner[5], Tomoko Ohyama[5], Emad Tajkhorshid[3], Donald J. van Meyel[2,4,5*], Renae M. Ryan[1*]

[1]School of Medical Sciences, Faculty of Medicine and Health, University of Sydney, New South Wales 2006, Australia.

[2] Department of Neurology and Neurosurgery and Integrated Program in Neuroscience, McGill University, Montreal, Quebec, Canada

[3]Theoretical and Computational Biophysics Group, NIH Center for Macromolecular Modeling and Bioinformatics, Beckman Institute for Advanced Science and Technology, Department of Biochemistry, and Center for Biophysics and Quantitative Biology, University of Illinois at Urbana-Champaign, Urbana, IL 61801, USA

[4]Research Institute of the McGill University Health Centre, Montreal, Quebec, Canada

[5]Department of Biology. McGill University, Montreal, QC, Canada.

[#]These authors share joint first authorship.

[*]Corresponding authors: renae.ryan@sydney.edu.au; don.vanmeyel@mcgill.ca

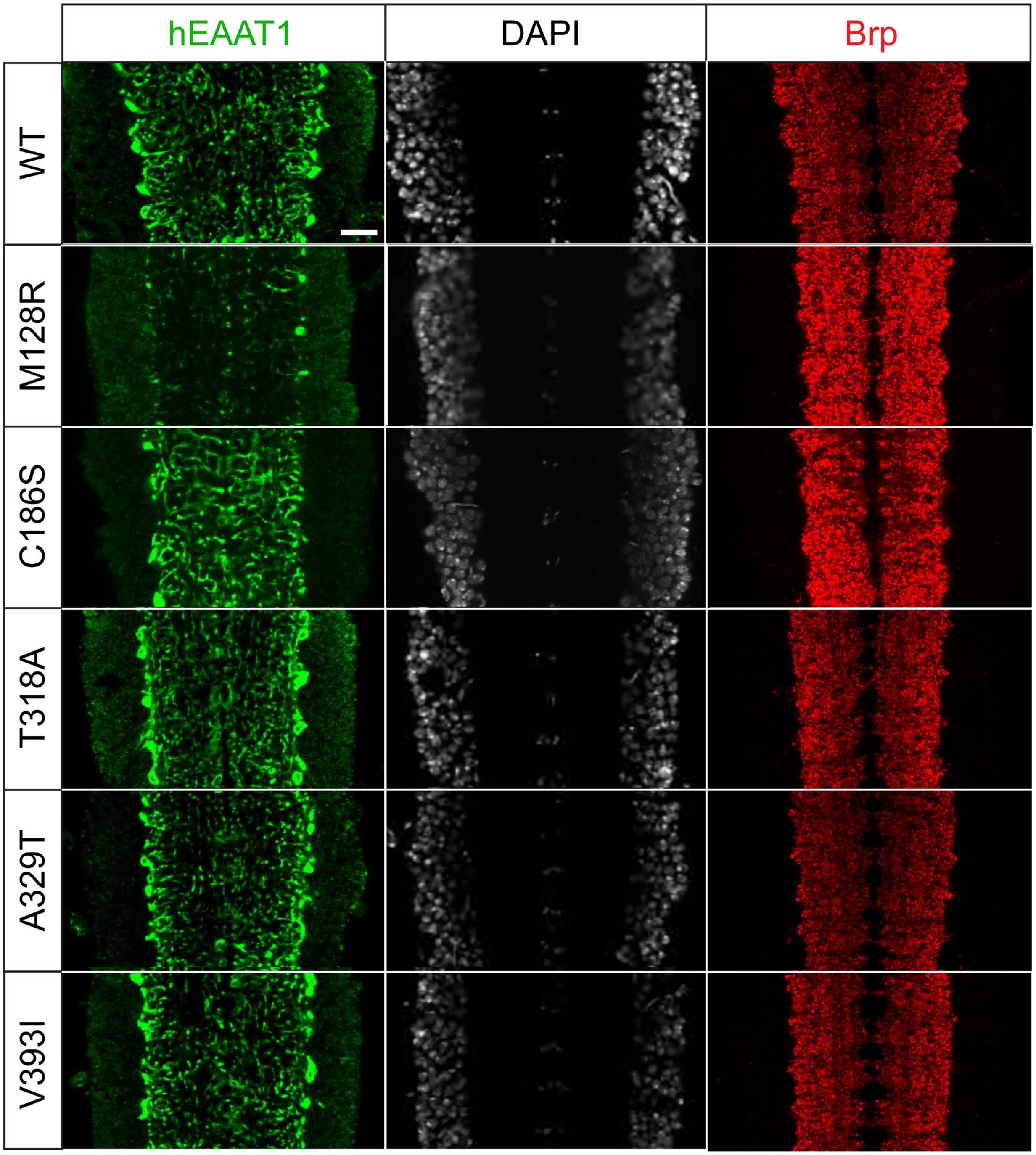

Supplementary Figure S1. EA6-related mutations do not cause neurodegeneration in first instar (L1) larvae. Representative image sets for each genotype show a single optical confocal section from the ventral nerve cord of a dissected larva labelled with immunohistochemistry for hEAAT1 (green), the nuclear stain DAPI (grayscale) or the synaptic protein Bruchpilot (Brp, red). Scale bar = 10 µm and applies to all panels. At least 5 animals were dissected, immunostained, and examined for each genotype. Like the control where *dEaat1*-null larvae are rescued with wild-type (WT) hEAAT1, all the EA6-related mutations of hEAAT1 except M128R are well expressed on the surface of infiltrative astrocyte processes within CNS neuropil. DAPI labeling of mostly neuronal nuclei in the cortex of the ventral nerve cord and Brp labeling of the synaptic neuropil, show that EA6-related mutations resemble controls and show no evidence for neurodegeneration.

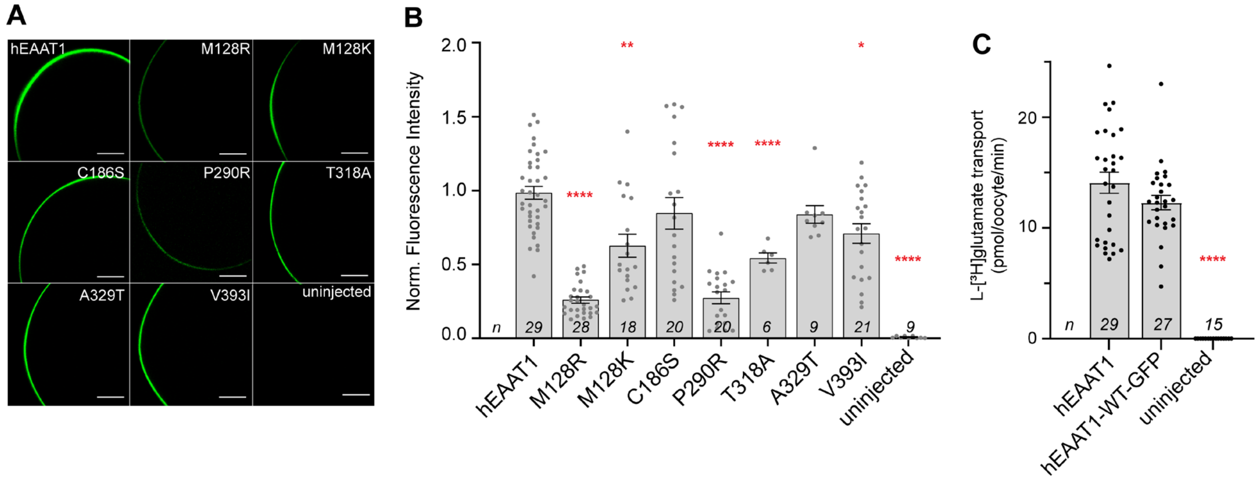

Supplementary Figure S2. **Surface expression of hEAAT1-WT and mutant transporters in *Xenopus laevis* oocytes. (A)** Representative images of oocytes expressing GFP-tagged hEAAT1-WT and mutant transporters are shown. Scale bars indicate 200 µm. **(B)** Average fluorescence intensity was measured along the cell surface to quantify relative expression levels between transporters. **(C)** Rates of L-[3H]glutamate transport were unaffected by addition of GFP tag. Values are normalised to the mean of wild-type values from the same batch of oocytes. Data represented as mean ± SEM, and the number of cells (n) used is indicated. Each dot represents the signal response from an individual cell. Experiments were conducted twice across two different batches of oocytes. One-way ANOVA tests (Brown-Forsythe) were performed for fluorescence intensity $F(8,79.75) = 32.23$, $p < 0.0001$, and L-[3H]glutamate uptake $F(2,48.7) = 94.78$, $p < 0.0001$.

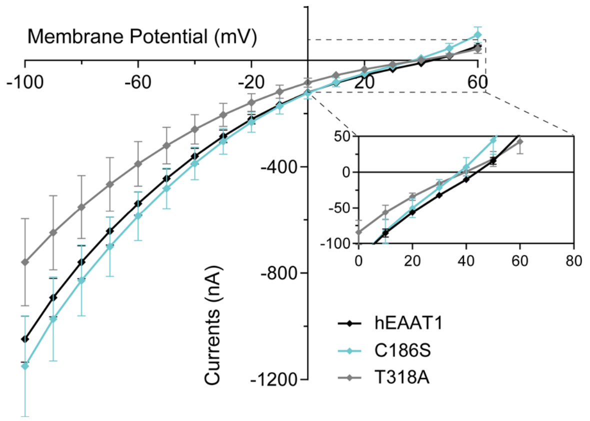

Supplementary Figure S3. Substrate activated Cl- conductance. Current-voltage (IV) relationships for hEAAT1, C186S and T318A, where reversal potentials (*Erev*) were analysed in **Fig. 4 B**. Currents were elicited by 100 µM L-aspartate in 10 mM Cl- buffer, in the presence of 100 mM Na+ (gluconate used as Cl- substituent to maintain osmolarity).

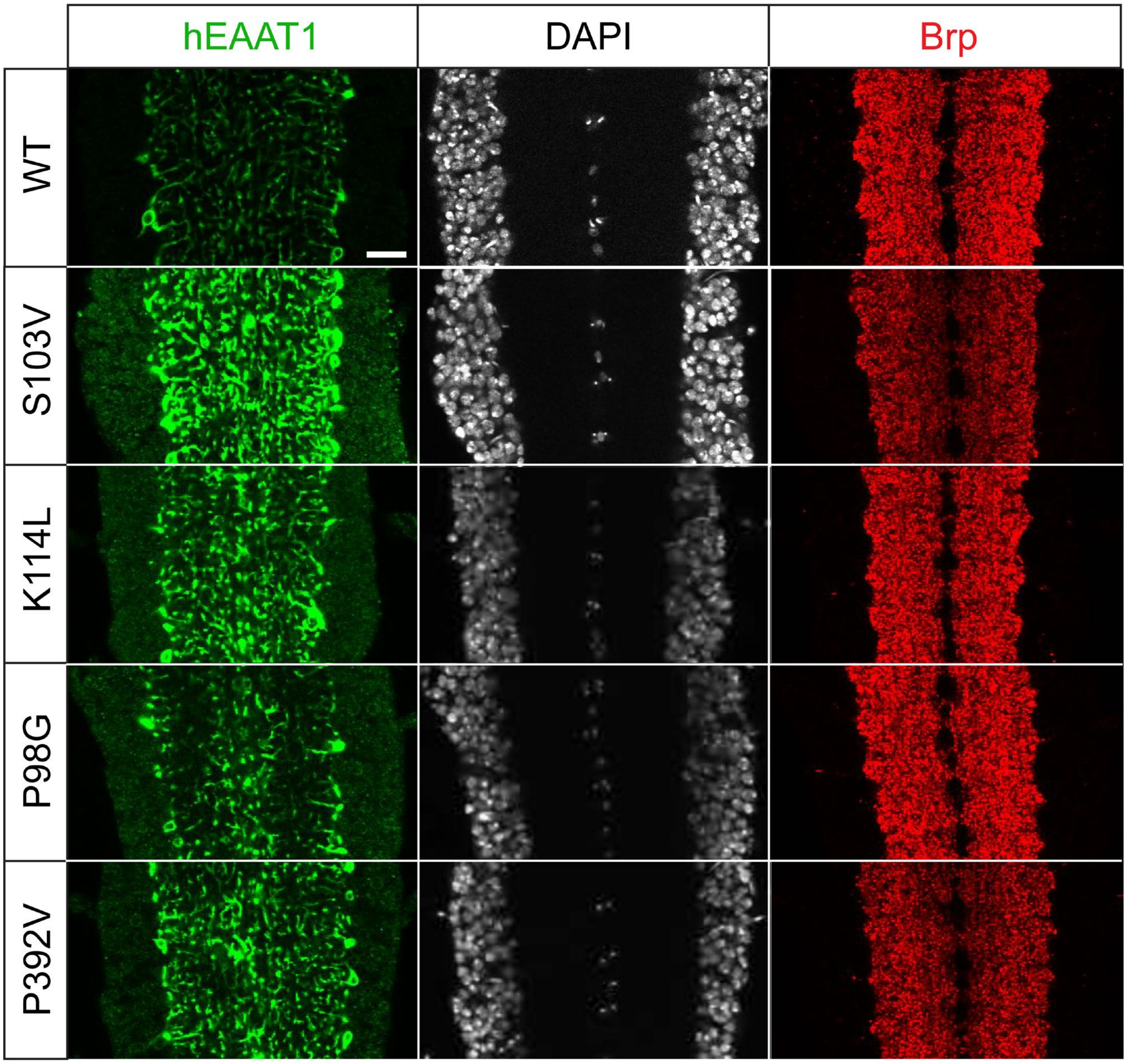

Supplementary Figure S4. **hEAAT1 Cl⁻ channel mutations do not cause neurodegeneration in L1 larvae.** Astrocyte-specific expression in *dEaat1*-null larvae of WT hEAAT1 or the mutations S103V, K114L, P98G, or P392V. Representative image sets for each genotype show a single optical confocal section from the ventral nerve cord of a dissected larva labelled with immunohistochemistry for hEAAT1 (green), DAPI (grayscale) or the synaptic protein Bruchpilot (Brp, red). At least 5 animals were dissected, immunostained, and examined for each genotype. Scale bar =10 µm and applies to all panels.

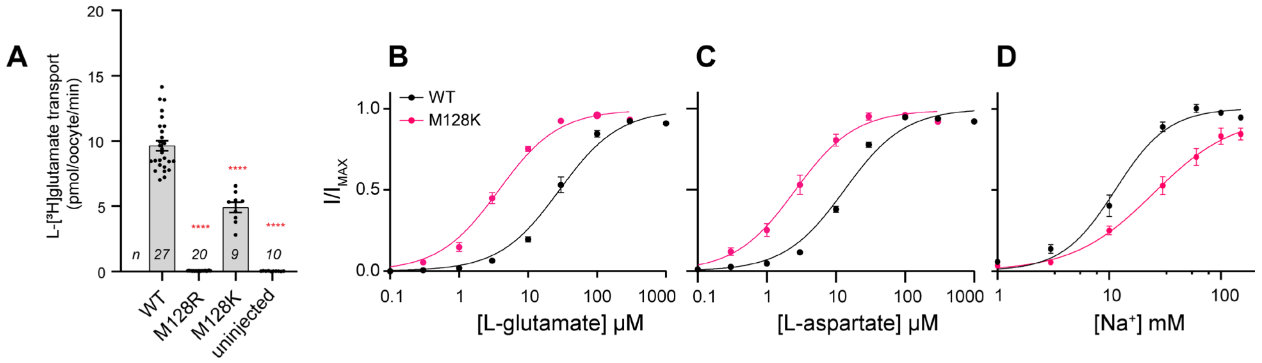

Supplementary Figure S5. M128K partially restores glutamate transporter function. (**A**) L-[^{3}H]-glutamate uptake, and dose-response relationships for L-glutamate (**B**), L-aspartate (**C**) and Na^{+} (**D**) for mutant transporter M128K are shown. Na^{+} titrations were performed with a saturating dose of L-glutamate (300 μM). Data for hEAAT1, M128R and uninjected cells from **Fig. 3 B-E** is provided for comparison. The number of cells (n) used is indicated in (A) and **Table 1** for panel B-D. One-way ANOVA tests (Brown-Forsythe) were performed for L-[^{3}H]glutamate uptake $F(3,31.8) = 374.7$, $p < 0.0001$.

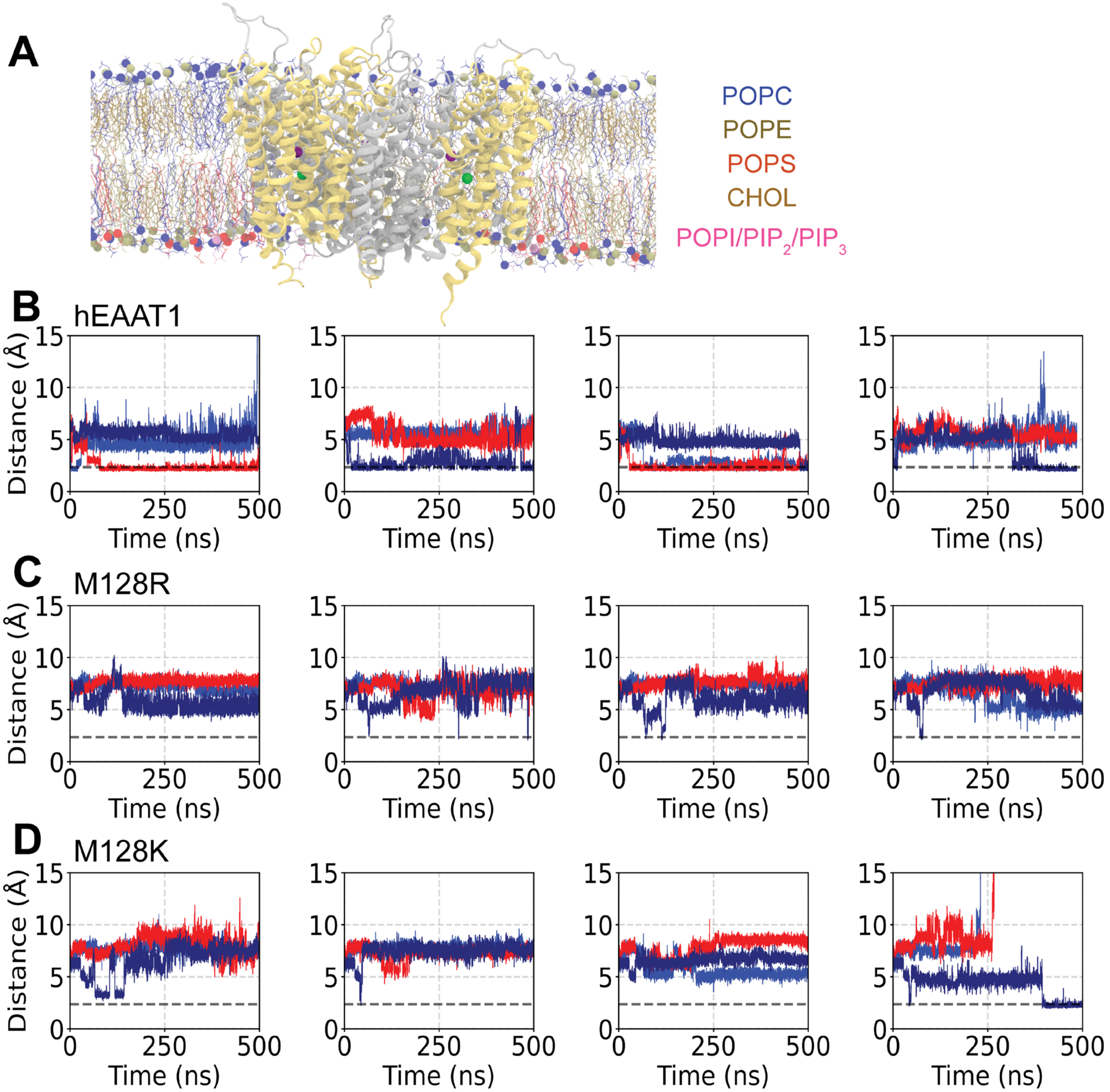

Supplementary Figure S6. Dynamics of Na+ migration in hEAAT1 and EA6 mutant transporters. (**A**) Initially the outward-facing partially bound conformation (Na+ occupying both Na1 (purple spheres) and Na2 (green spheres) sites, with aspartate bound) of hEAAT1, in a trimeric state, was embedded into an asymmetric lipid bilayer containing POPC (tan), POPE (grey), POPS (red), Cholesterol (cyan), POPI (ice blue), and small quantities of PIP1, PIP2, and PIP3 (grey) lipids. The scaffold and transport domains of the transporter are shown in cartoon representation in silver and gold, respectively. Four independent simulations were run in each system. We monitored the time evolution of Na+ binding to the Na3 site by calculating the distance between the ion initially in Na1 and D400, a negative side chain that coordinates the ion in Na3. (**B**) Multiple Na+ hopping events are captured in hEAAT1, where the Na+ ion from Na1 translocated to Na3 (dashed line). (**C**) In M128R, no movement of Na+ to Na3 is observed. (**D**) In M128K, only one single hopping event was captured.

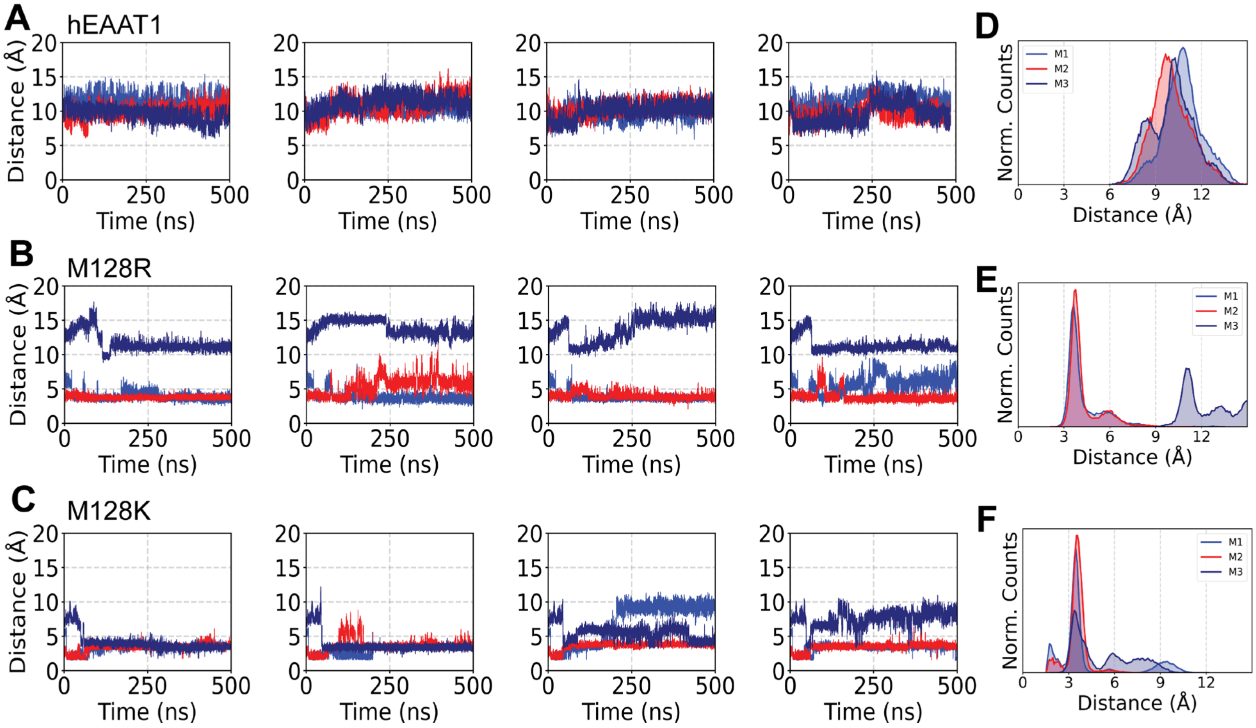

Supplementary Figure S7. The basic side chains of M128R and M128K can interact with D400. (**A-C**) Time series of the minimum distance between any side chain oxygen atom of D400 and either the S atom in M128 (**A**), or terminal hydrogens in M128R (**B**) or M128K (**C**) was monitored to represent the proximity of the side chains. Four independent simulations were run in each system, and data for each protomer in each simulation are plotted with a different color. (**D-F**) The distribution histograms of the distances for each protomer (M1-3) in the three systems are shown. (**D**) M128 in all three hEAAT1 protomers adopts an 'OUT' conformation and does not come in proximity to D400, as indicated by all distance distributions longer than 7 Å. (**E**) The arginine side chain in one of the three protomers of M128R also adopts an 'OUT' conformation (M3), while in the other two (M1, M2) it adopts an 'IN' confirmation and forms a salt-bridge with D400. (**F**) The lysine side chain of M128K fluctuates between the two states, with a higher propensity for an 'IN' confirmation. Overall, the presence of a basic side chain at position 128 (M128R or M128K) substantially increases the probability of salt-bridge formation with D400 as illustrated by the distance distributions in **E** and **F**.

Movies 1–11. 10x speed reconstructions of infrared tracking of L1 larvae over 180 s, demonstrating lack of larval crawling in dEaat1 null animals (Movie 1), rescue of larval crawling by expression of hEAAT1 in larval astrocytes with alrm-Gal4 (Movie 2), and varying degrees of rescue with each of the EA6-related hEAAT1 mutations M128R, C186S, T318A, A329T and V393I (Movies 3-7) and the additional mutations that affect Cl- channel function S103V, K114L, P98G and P392V (Movies 8-11)

Movie 12. Na^+ migration from the Na1 site to the Na3 site in hEAAT1. Starting from the outward-facing partially-bound conformation of EAAT1 (shown in gold, cartoon representation), spontaneous hopping of Na^+ (green sphere) from the Na1 site to the Na3 site (residues D487 (Na1), D400 (Na3) and M128 shown in stick representation).

Movie 13. Membrane deformation caused by the M128R mutation. The arginine sidechain (stick representation) at position 128 is frequently observed to adopt an orientation facing the lipids, which results in recruitment of lipid headgroups (stick representation with carbon atoms in green) and bilayer deformation (phosphorous atoms are shown as light brown spheres).
